# Bile Acid Flux Is Necessary for Normal Liver Regeneration

**DOI:** 10.1371/journal.pone.0097426

**Published:** 2014-05-19

**Authors:** Willscott E. Naugler

**Affiliations:** 1 Dept. of Medicine, Division of GI & Hepatology, Oregon Health & Science Center, Portland, Oregon, United States of America; 2 Oregon Stem Cell Center, Oregon Health & Science Center, Portland, Oregon, United States of America; Beckman Research Institute of City of Hope, United States of America

## Abstract

**Background & Aims:**

Many signals governing liver regeneration (LR) following 2/3 partial hepatectomy (PH) are recognized, but the primary signal(s) remains unknown. The aim of the study was to confirm that the remnant liver after PH lacks capacity to secrete the BA pool returning via the enterohepatic ciruculation (EHC), which may in turn stimulate LR.

**Methods:**

After standard PH, BA flux was documented and BA signaling (Fgf15) and synthesis (Cyp7a) determined by qPCR. Rat biliary fistula (BF) and Asbt knockout mouse models interrupted the EHC prior to PH, and standard assays for LR employed along with complete RNA sequencing. CCl_4_ intoxication after BF tested the hypothesis in an alternate injury model.

**Results:**

BA rise in systemic blood immediately following PH, confirming that the remnant liver cannot handle the BA returning via portal circulation. When the BA pool is drained prior to PH in the rat BF model, LR is markedly attenuated, a phenomenon reversed with duodenal BA replacement. Hepatocyte proliferation is similarly attenuated after PH in the Asbt knockout mouse as well as after CCl4_4_ intoxication in rats with BF. Complete RNA sequencing in the rat PH model shows that early c-jun and AP-1 gene expression pathways are down regulated in the absence of BA, coincident with attenuated LR.

**Conclusions:**

Absent BA return to the liver after PH or CCl_4_ injury markedly attenuates LR, though hepatocyte proliferation still occurs, inferring that BA flux and signaling are not the sole signals governing LR. Transcriptional networks involving c-jun and AP-1 are involved in the BA-specific effects on hepatocyte proliferation.

## Introduction

Liver regeneration following 2/3 partial hepatectomy (PH) is a well-described phenomenon that results in restoration of liver mass in the rodent 7-10 days after PH [1], [2]. PH is followed by the coordinated replication of all liver cell types, beginning with hepatocytes and followed by the various non-parenchymal cells [3] until liver mass is restored. Many signals associated with liver regeneration have been described, and include both humoral and intra-cellular signaling, as well as potential reliance on non-parenchymal cell involvement for successful completion of the process [4]. Deletion or knock-down of most described pathways often results in significant delays in regeneration, but no single signal has been identified that is both necessary and sufficient to drive liver regeneration. Amongst the most important questions regarding the phenomenon are (1) what are the earliest and most important signals that initiate liver regeneration, and (2) what are the mechanisms and signals that govern termination of the process? The latter refers indirectly to regulation of liver size, with reference to the aptly named “hepatostat,” [5] a concept suggesting liver size is regulated by some physiological function(s). Amongst physiologic functions of the liver, multiple lines of study have demonstrated involvement of glucose, fat, and protein homeostasis in regulation of liver regeneration [6], though specific mechanisms remain elusive. Finally, there is the thorny “cell of origin” question for proliferating hepatocytes in various injury and regeneration models, but at least for the PH model, the adult hepatocyte itself seems to be the primary source of new hepatocytes [7].

Bile acid (BA) homeostasis has recently gained some traction as a contributor to liver regeneration. Decades ago it was first noted that external biliary drainage led to a decrease in regenerative capacity,[8] while internal biliary drainage did not [9]. BA levels were not specifically measured in these studies. Cholestyramine (BA binding-resin) fed mice restored liver mass to only 50% of controls after PH, [10] and a study of human surgical liver resections revealed an association between serum BA levels and liver regeneration [11]. Farnesoid X Receptor (FXR) is a nuclear receptor whose primary ligands are BA, and FXR-/- mice [12] display significantly impaired liver regeneration after PH [13]. Further studies with liver- and intestine-specific FXR knockouts revealed that this nuclear receptor had both hepatocyte and intestinal contributions to liver regeneration after PH [14]. Lastly, recent studies have shown that mice deficient in the intestinal negative feedback signal for BA, FGF 15, had a markedly abnormal response to PH with significant mortality [15], and mice deficient in the membrane BA receptor, TGR5, demonstrated hepatic necrosis after PH due to BA overload [16].

The present study sought to clarify the physiology of bile acid flux after PH with attention to relevant signaling in both liver and intestine. In addition, we tested the hypothesis that bile acid flux after PH is necessary for initiation of hepatocyte proliferation and restoration of hepatic mass. In the rat biliary fistula model, we show that drainage of the BA pool prior to PH results in decreased hepatocyte proliferation and decreased restoration of liver mass after 7 days, and that these findings can be reversed with infusion of physiological levels of BA into the intestine. RNA sequencing of liver samples in this model shows a BA-specific decrease in JNK and AP-1 signaling pathways 4 hours after PH, as well as FOXM1 at 24 hours. We showed similar findings of decreased regeneration in Asbt deficient (Asbt-/-) mice lacking the intestinal BA transporter, thus producing a alternate model of interrupted enterohepatic circulation (EHC). In addition, we show that CCl_4_ injury in the rat biliary fistula model results in similar levels of hepatic injury, but reduced hepatocyte proliferation, a finding that can be reversed with BA infusion. In summary, this study presents evidence that BA flux is necessary for normal liver regeneration, but is not the only factor in initiation and regrowth of the liver after PH.

## Materials and Methods

Full methods are available in Materials and Methods S1.

### Animal Studies

C57BL/6 male mice (8 weeks old) from Jackson Laboratories were used to describe bile acid flux after partial hepatectomy. Male Sprague Dawley rats (4–5 months old) from Charles River were used for the biliary fistula studies. Asbt deficient mice (Asbt-/-) were a kind gift from Dr. Paul Dawson [17]. All studies were approved by the Oregon Health & Science University Institutional Animal Care (OHSU IACUC #IS00000493) and Use Committee as set forth in the Guide for the Care and Use of Laboratory Animals published by the National Institutes of Health. All surgery was performed under isoflurane anesthesia with post-operative pain control using buprenorphine and/or meloxicam and all efforts were made to minimize suffering.

### Biliary fistula (BF) and bile acid (BA) infusion models

A BF was created by placing a catheter in the common bile duct as previously described.[18] At the same time an enterostomy was created and a catheter placed in the duodenum just proximal to the insertion of the common bile duct. All rats (including controls who received sham biliary fistulas) got enterostomy catheters for infusion of fluids. Both catheters were externalized. All rats received continuous duodenal infusions of normal saline throughout all experiments. For the BF + BA infusion, sodium taurocholate (NaTCA, Sigma, Cat # T4009) was infused at a rate of 0.5 mg NaTCA/gram body weight/day, which corresponds to the rate empirically determined by measuring total BA coming from the BF of rats (collected for the first 4 hours after BF placement). Pulse or continuous BrdU (Sigma, Cat # B9285) was given through the duodenal catheters. Figure 2A notes the experimental design and timecourse.

**Figure 1:**
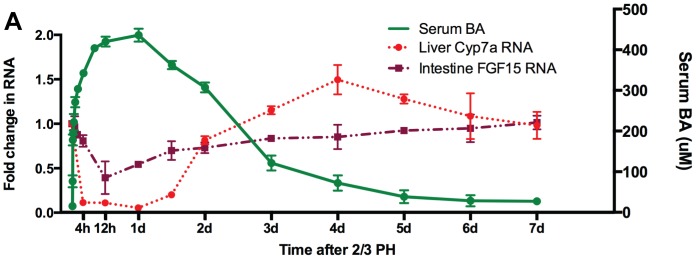
Bile acid (BA) flux and hepatocyte proliferation after 2/3 partial hepatectomy (PH) in mouse. PH was performed in mice, and blood, liver and intestines were examined over a 7 day timecourse (3 mice per timepoint). Systemic serum BA rise immediately after PH indicating that the remnant 1/3 liver cannot handle the BA returning via enterohepatic circulation (EHC) from the intestine, thus moving from the EHC to the blood compartment. Liver Cyp7a is repressed soon after PH, followed later by a decrease in FGF15 RNA from the intestine.

**Figure 2:**
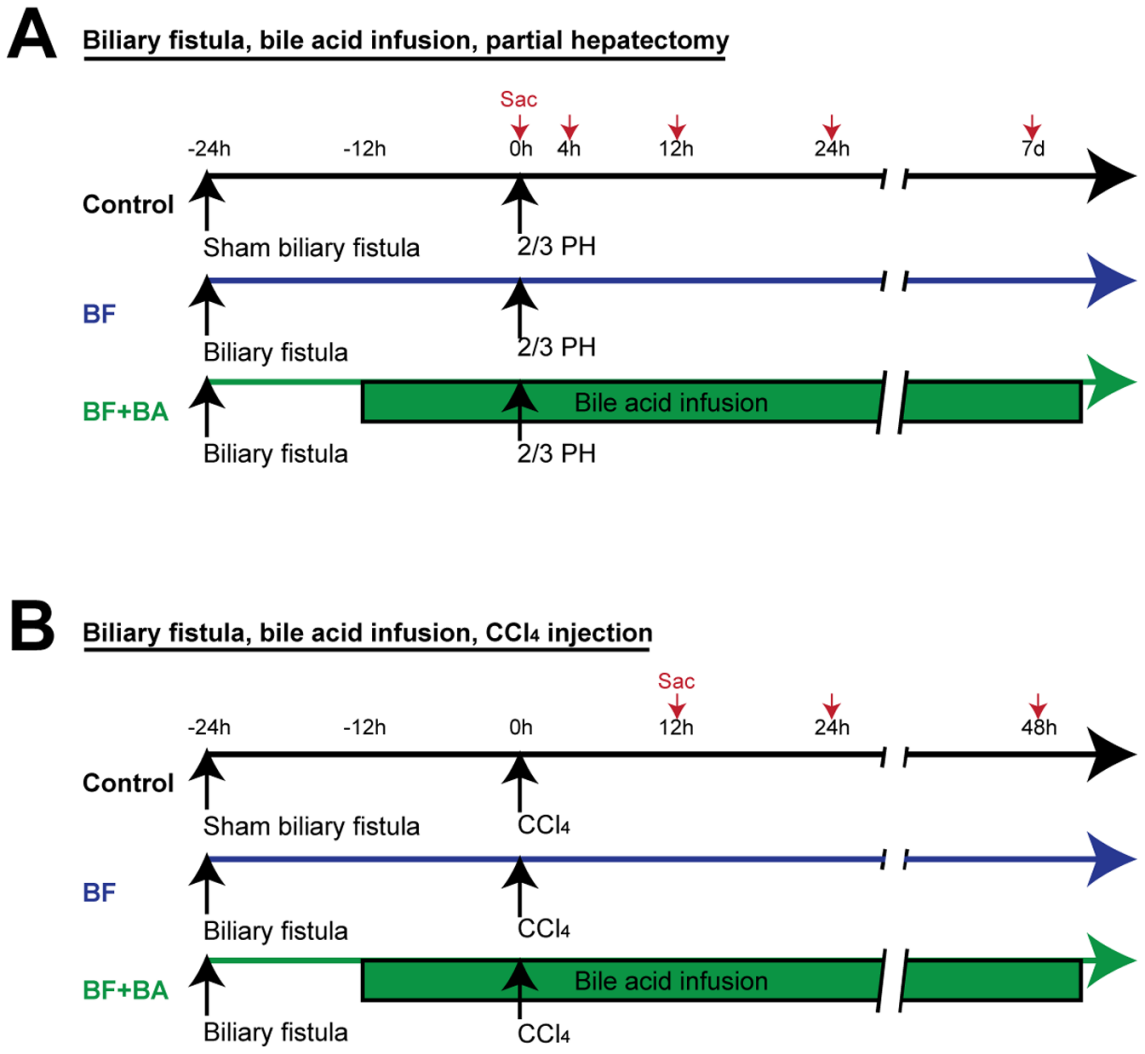
Outline of biliary fistula, BA replacement rat model for PH and CCl_4_ injury. Male Sprague-Dawley rats received biliary fistula (or sham) along with duodenal catheter placement and were allowed to recover for 24 hours prior to (A) PH or (B) CCl_4_. The intestinal BA pool would therefore be drained prior to PH or CCl_4_. In the “BF+BA” group, Na taurocholate was infused via the duodenal catheter 12 hours prior to PH or CCl_4_, replacing the intestinal BA pool (but not other constituents of bile) before injury.

### Partial hepatectomy (PH) and CCl_4_ intoxication

In rat experiments, 2/3 PH was performed as described [1], with a midline incision distant from the left lateral incision used to perform the prior BF surgery. PH in the Asbt-/- mice followed a modified protocol [19]. For CCl_4_ experiments, CCl_4_ 2 mL/kg in a 20% solution in corn oil was given via the duodenal catheter. After above liver injuries, liver, intestine, and serum were collected at indicated times. Figure 2B notes the experimental design and timecourse.

### Tissue analysis

Liver, intestine and serum were snap frozen in liquid nitrogen when animals were killed; some liver was fixed in formalin for immunohistochemistry. Quantitative real-time RT-PCR (qPCR), complete RNA sequencing, serum bile acid, HGF and IL-6 levels were determined as noted in Materials and Methods S1. All qPCR experiments results were normalized to the zerotimepoint, and internally controlled for RNA quantity with the housekeeping gene Gapdh (mouse or rat, primers noted in Table S1 in File S1).

Raw RNA sequence data available on the NCBI GEO website, series record GSE54673, http://www.ncbi.nlm.nih.gov/geo/query/acc.cgi?acc=GSE54673, also noted in Table S3 in File S1. Gene set constituents are noted in Table S2 in File S1. RNA quality for the RNA sequencing is noted in Figure S2.

### Statistical Analysis

Prism 6 (GraphPad Prism) was used to graph all noted data except for RNA sequence data (see Materials and Methods S1). Results are expressed as mean values + SD. P values were calculated by two-sided independent t-test, and a p value<0.05 was considered significant.

## Results

### Systemic serum BA rise sharply immediately following PH along with dynamic enterohepatic BA signaling

To clarify the physiology of BA flux within the enterohepatic circulation (EHC) following PH, we performed PH [19] on male C57/B6 mice and collected systemic blood, liver, and ileum at various timepoints over a 7-day period. We measured total BA in serum, and mRNA levels of Cyp7a in the liver and Fgf15 in the intestine. Cyp7a is the primary rate-limiting enzyme controlling BA synthesis from cholesterol [20], and Fgf15 is the intestinal cytokine that provides feedback inhibition of hepatic BA synthesis through down-regulation of Cyp7a [21]. Serum BA rise quickly after PH (Figure 1), indicating failure of the remnant liver to take up and secrete the BA load returning from the intestine. This is not surprising, given that the large majority of the BA pool resides at any given time in the intestine, and BA return from the intestine would continue at the same rate compared to before PH. Liver has a finite secretory capacity for BA [22], thus when the remnant 1/3 liver after PH suddenly encounters 3 times the normal BA inflow, BA return beyond the secretory capacity of the liver spill into the systemic circulation. Similar findings have been reported previously in mice [23] and rats [23]–[25]. Liver Cyp7a mRNA levels fall precipitously after PH, followed later by a decrease in the intestine Fgf15 (Figure 1). This clearly indicates that an increase in Fgf15 expression is not responsible for the decrease in Cyp7a.

Administration of BrdU 2 hours prior to killing mice labels proliferating cells in the liver, and typical hepatocyte proliferation was noted in these mice with a peak proliferation about 48 hours after PH (Figures S1A and S1B). These results indicate that the remnant liver after PH does not have the capacity to secrete the BA load returning via the portal circulation, and that failure of this function is evident within minutes after PH, a time coincident with the earliest signals leading to hepatocyte proliferation.

### Removal of the BA pool attenuates liver regeneration after PH, and infusion of BA specifically restores regeneration

**Figure 3:**
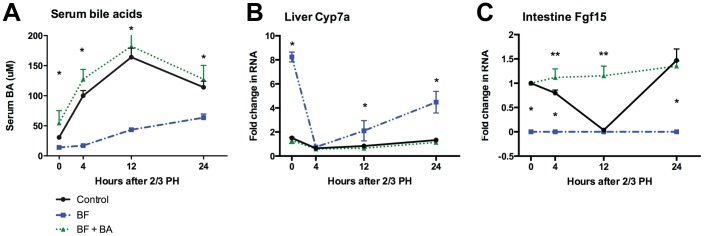
Bile acid flux and signaling after PH in biliary fistula and BA replacement model. Rats underwent sham biliary fistula (Control) or biliary fistula (BF) 24 hours prior to PH. Comparisons of serum bile acids (A), liver Cyp7a (B) and intestine Fgf15 (C) are shown between the experimental conditions (n = 4 rats per timepoint). A single asterisk (*) indicates a significant (p<0.05) difference between the control/BA replacement and the biliary fistula, while a double asterisk (**) indicates a significant difference between the control and the BA replacement.

To drain the BA pool, a rat model of biliary fistula (BF) was used (Figure 2A) wherein bile was drained for 24 hours prior to PH. Control rats (sham BF) showed similar changes in serum BA, liver Cyp7a and intestine Fgf15 (Figures 3A–C) compared to mice. As expected, rats that had undergone BF for 24 hours prior to PH had elevated Cyp7a levels (Figure 3B). Fgf15 levels remained undetectable throughout the timecourse as would be expected with no BA in the intestine due to the BF (Figure 3C). The BF markedly suppressed the post-PH rise in serum BA at 4 hours, but they slowly began to rise, along with Cyp7a levels at 12 and 24 hours post-PH. These data indicate that hepatocytes begin synthesizing *de novo* BA at a higher rate than they can secrete beginning 12 hours after PH. Interestingly, there is again a marked suppression of Cyp7a mRNA soon after PH in the BF condition, indicating that the suppression is caused by neither Fgf15 nor high systemic BA levels, and must be due to an as-yet unidentified mechanism. Changes in BA flux, Cyp7a and Fgf15 noted in the BF model could all be reversed with the duodenal infusion of Na-Taurocholate (NaTCA). Data is presented per experimental condition (rather than tested variable) in Fig. S5 A–C.

**Figure 4:**
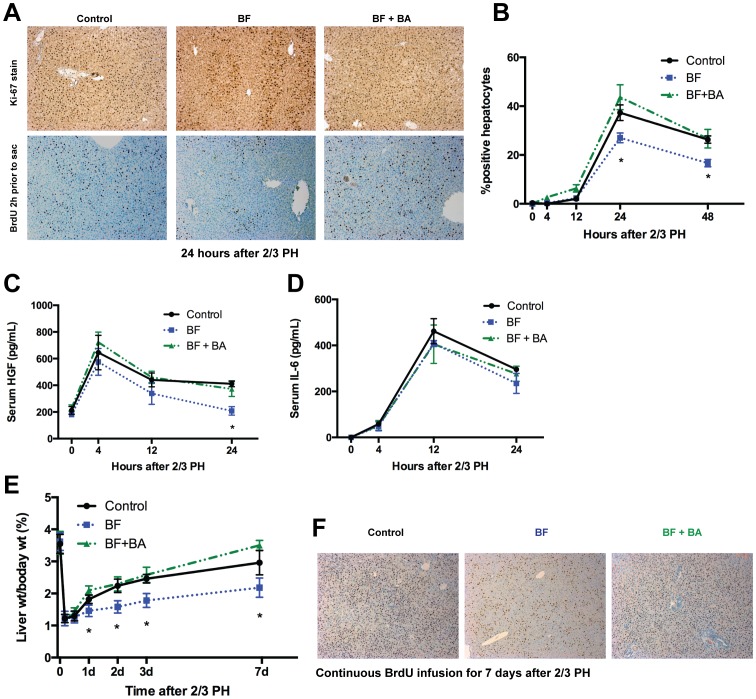
Liver regeneration after PH in biliary fistula and BA replacement model. Hepatocyte proliferation 24(A,B) by either Ki-67 or BrdU staining and counting of positive hepatocyte nuclei. Hepatocyte proliferation is significantly (but not completely) diminished in the BF, and restored when BA infusion is added to the BF. Serum levels of HGF are diminished (C) 12–24 hours after PH in the BF model, but there are no significant differences between models in serum IL-6 levels (D). Figures 4A–D represent 4 rats per timepoint. When followed for 7 days after PH, rats BA drainage have significantly smaller livers (E), and less BrdU staining when BrdU is continuously infused throughout the 7 days after PH (F). Figures 4E–F represent 3 rats per timepoint. A single asterisk (*) indicates a significant (p<0.05) difference between the control/BA replacement and the biliary fistula.

Assessment of hepatocyte proliferation after PH by either Ki-67 or Brdu staining (Brdu given 2 hours prior to liver collection) revealed the normal marked increase in proliferation in control rats at 24 hours, but a significant decrease in proliferating hepatocytes in rats with BF (Figures 4A & 4B). This deficit in proliferating hepatocytes could be restored to normal with the duodenal infusion of NaTCA in rats with BF. These data indicate that draining of the BA pool attenuates hepatocyte proliferation after PH as has previously been shown,[9]–[11] but also that the effect is specific to BA, thus indicating that BA return to the regenerating liver is required for normal liver regeneration. Serum HGF levels (Figure 4C) rise after PH even in the absence of BA, but the levels are significantly lower in later time points. Serum IL-6 levels (Figure 4D) rose after PH and levels did not differ whether BA were present or not. These data are congruent with reports of HGF being necessary for liver regeneration [26]–[28], while IL-6 is probably dispensable [29].

Non-parenchymal cell (NPC) proliferation occurs after hepatocyte proliferation during liver regeneration after PH [3]. No significant NPC proliferation (by BrdU-labeling) was seen until 3 days after PH in the rat (data not shown). Fewer BrdU-labeled NPCs were noted at day 3 and day 7 of the continuous BrdU infusion experiment (noted in Figure 4F, but NPC data not quantified).

Like hepatocyte proliferation, liver regrowth after PH is significantly attenuated in rats with prior BF, a finding reversed with BA infusion (Figure 4E). Continuous BrdU infusion after PH over 7 days (Figure 4F) reveals that nearly all hepatocytes in control rats have undergone replication, while this number is significantly fewer in rats with BF, again a finding which is reversed with intestinal BA infusion in BF rats.

Taken together, these data show that BA flux is necessary for normal hepatocyte proliferation and regrowth of the liver after PH.

### AP-1 and Hippo gene regulation pathways down-regulated after PH in rats with depleted BA pool

**Figure 5:**
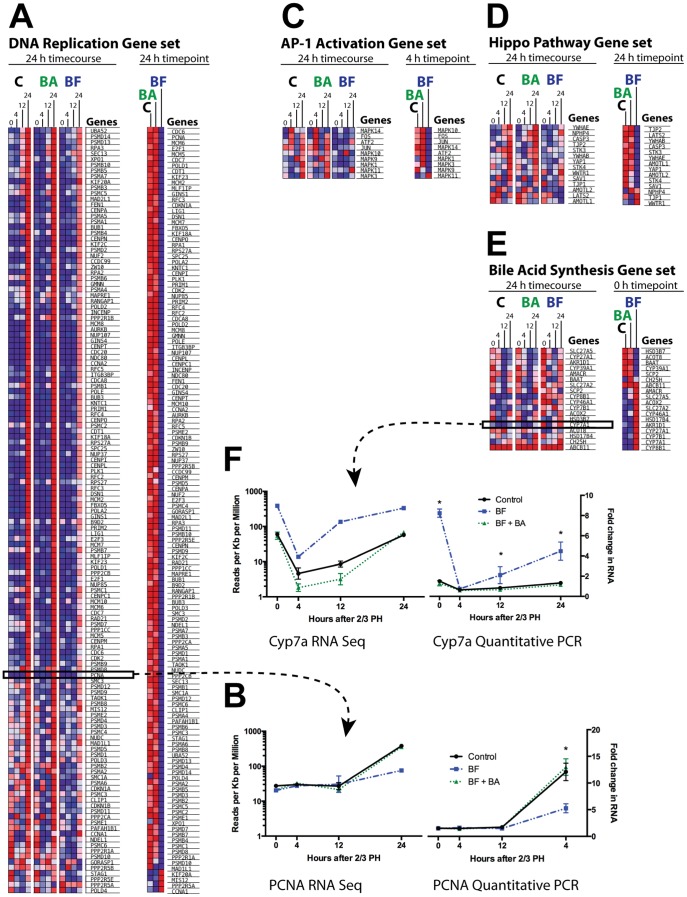
RNA sequencing of liver after PH in rats with BF and BA infusion. Total RNA from liver of four rats per group/timepoint had complete RNA sequencing. Pathway analysis revealed an increase in gene expression related to DNA replication (A) in all groups at 24 hours post-PH, but significantly less at the 24 hour timepoint in the BF group compared to control and BF + BA infusion groups. Gene expression in the AP-1 pathway was increased at 4 hr post-PH in the control and BF + BA infusion groups (C), but the BF group had markedly and significantly decreased 4 hr expression compared to the other two groups. Gene expression in the Hippos pathway increased in all groups at 24 hours (D), but significantly less in the BF group. As expected the time zero bile acid gene expression pathway was increased compared to control and BF + BA infusion groups (E). Quantitative PCR for PCNA (B) and Cyp7a (F) confirmed similar patterns in these genes compared to the RNA sequencing data. A single asterisk (*) indicates a significant (p<0.05) difference between the control/BA replacement and the biliary fistula.

Whole liver tissue was collected at 0, 4, 12, and 24 hours after PH in rats with sham BF (controls), BF, and BF with duodenal BA infusions, and RNA extracted and sent for complete RNA sequencing (4 samples per timepoint per condition). Pathway analysis revealed up-regulation of genes involved in DNA replication at 24 hours (Figure 5A) in all conditions (“24 h timecourse”). When samples from the 24 hour timepoint only were compared, however, genes involved in DNA replication were significantly less up-regulated in rats with BF compared to control and BA infusion rats (“24 h timepoint”). This data is congruent with the histological findings of less hepatocyte proliferation 24 hours post-PH.

The AP-1 transcriptional pathway has been shown to drive cyclin D1 expression and proliferation after PH,[30] and a major upstream kinase of this pathway, c-jun-N-terminal kinase (JNK), is strongly activated within minutes after PH [31]. In addition, BA are known to activate JNK in hepatocytes [32], [33]. We found that genes associated with AP-1 activation were significantly up-regulated 4 hours after PH in control rats (Figure 5C), which was nearly abolished in rats with BF and restored with BA infusion. This data suggests that BA are primary activators of JNK and the AP-1 gene pathway after PH. Western blotting for phosphor-JNK (the activated form of JNK) showed a bimodal activation in control and BA-replenished rats after PH, and a clear decrease in this activation in BA-depleted rats (Figure S4).

Though the primary physiologic factors controlling liver size are unknown, it is clear that liver size is at least partially controlled at the transcriptional level by the Hippo pathway. Mice with overexpression of YAP, mammalian effector of the Hippo pathway, develop massively enlarged livers [34], [35]. We found that the Hippo pathway was significantly upregulated at 24 hours after PH in control rats and those with BF plus BA replacement (Figure 5D); rats with BF and depleted BA pools, however, had significantly less activation of the Hippo pathway at 24 hours, congruent with later findings of diminished liver growth in rats with BF.

Not surprisingly, there were marked time zero differences in expression of genes governing BA synthesis (Figure 5E) between rats with BF and controls; these differences were largely erased by duodenal BA infusion. As expected, there were significant differences at various timepoints in the BA-associated Fxr and Shp (Figure S3B, S3C) as well as proliferation-associated Foxm1 (Figure S3D).We performed qPCR on several genes (Cyp7a and PCNA shown) and compared these data to that obtained with RNA sequencing (Figures 5B and 5F). Both methods showed similar results for RNA expression.

### Liver regeneration is attenuated in Asbt-/- mice

**Figure 6:**
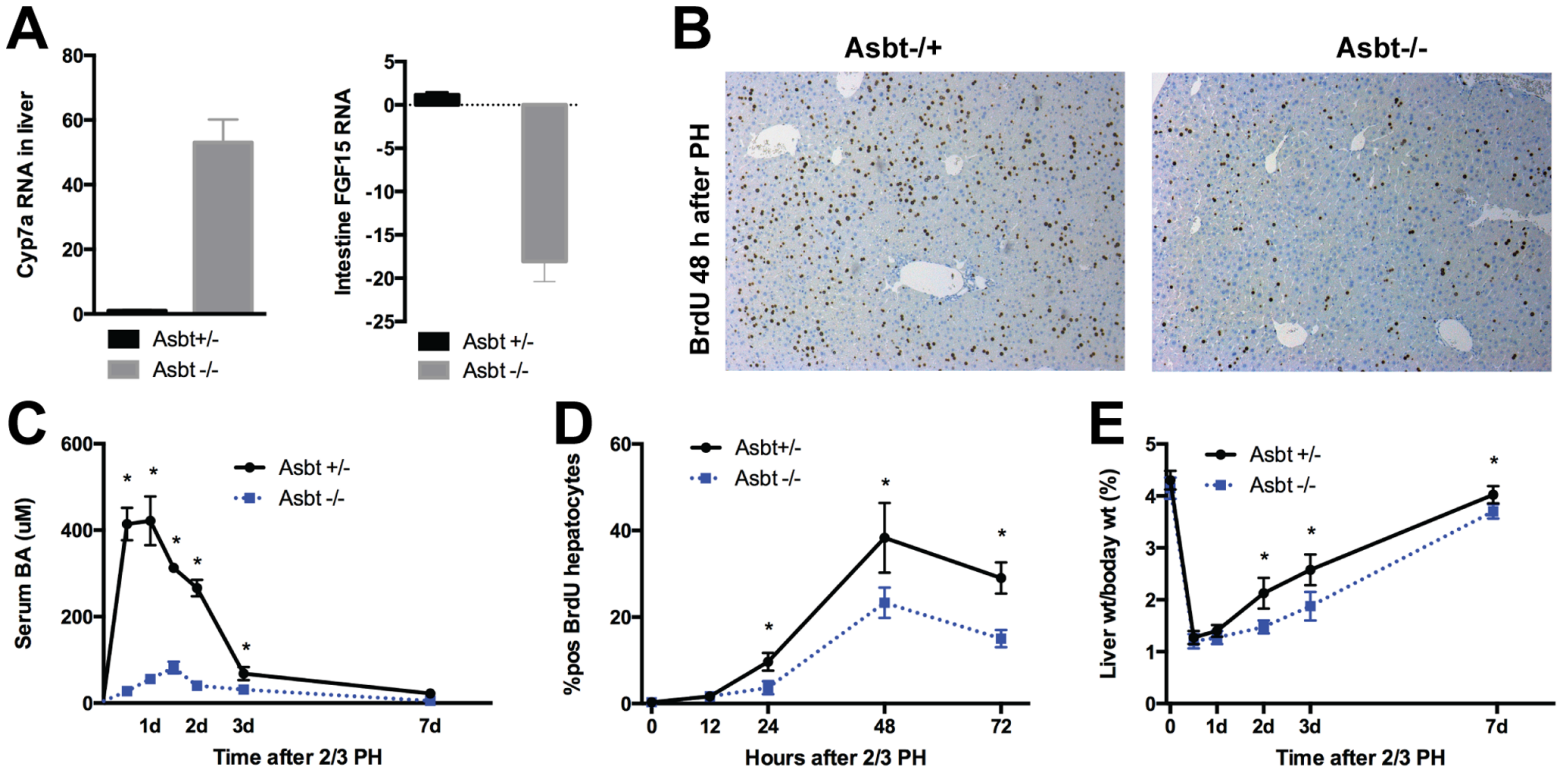
Liver regeneration is attenuated in Asbt-/- mice. Asbt-/- mice lack the intestinal BA receptor, thus interrupting the EHC and resulting in no portal venous return of BA. As previously described, Asbt deficiency results in marked upregulation of liver Cyp7a (A) due to absence of negative feedback from intestinal FGF15. Hepatocyte proliferation after PH is significantly attenuated (B, D) as noted on BrdU staining. Serum BA levels increase significantly less (C) in Asbt-/- mice after PH, indicating an absence of portal venous return of BA. Liver regrowth is initially attenuated (E) in Asbt-/- mice after PH. 3 mice per timepoint were used in these experiments. A single asterisk (*) indicates a significant (p<0.05) difference between the groups.

Asbt is the intestinal transporter on the luminal side of enterocytes responsible for intestinal uptake of BA. ASTB -/- mice therefore do not absorb BA from the intestine and thus have an interrupted EHC [17]. Asbt-/- mice have markedly elevated Cyp7a levels in the liver and nearly undetectable Fgf15 RNA in the intestine (Figure 6A) compared to their heterozygote littermates. After PH, Asbt-/+ mice have normal levels of hepatocyte proliferation at the peak 48 hour timepoint, but homozygous knockouts have significantly fewer hepatocytes undergoing replication (Figures 6B and 6D). As expected, the early and significant rise in serum BA after PH is significantly blunted in Asbt-/- mice compared to their heterozygous littermates (Figure 6C). Liver growth is diminished after PH in Asbt-/- mice, and is significantly smaller compared to controls at all time points (Figure 6E). Of note, however, there is still hepatocyte proliferation (though less) in Asbt-/- mice after PH, and there is still liver regrowth after PH. Together, this data presents another model wherein BA return to the liver is abolished, and correlates this with diminished regenerative response to PH in the liver.

### Hepatocyte proliferation, but not injury, is significantly diminished in rats with BF prior to CCl_4_ intoxication

**Figure 7:**
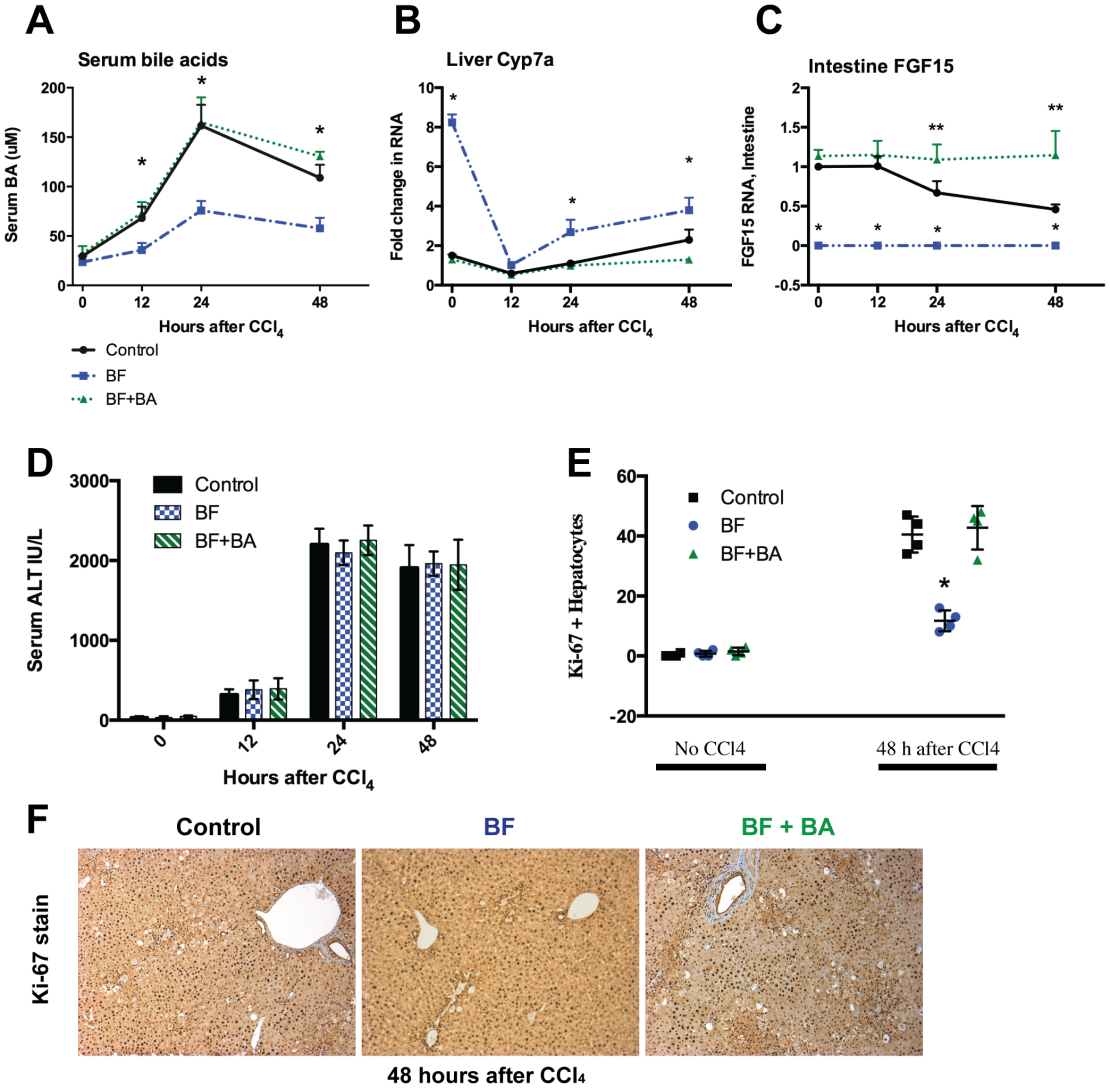
Hepatocyte proliferation is significantly reduced in rats with BF after CCl_4_ intoxication. After sham BF (controls), BF, or BF + BA infusion, rats were given 2 mg/kg CCl_4_, and assessed afterwards. Comparisons of serum bile acids (A), liver Cyp7a (B) and intestine Fgf15 (C) are shown between the experimental conditions. Serum ALT levels after CCl_4_ were similar in all groups when used to quantitate liver injury (D), but hepatocyte proliferation was attenuated in rats with BF compared to controls when assessed by Ki-67 staining (E,F). Proliferation was restored when a BA infusion was given to rats with a BF. Four rats per timepoint were used in these experiments. A single asterisk (*) indicates a significant (p<0.05) difference between the control/BA replacement and the biliary fistula experiments, while a double asterisk (**) indicates a significant difference between the control and the BA replacement.

PH is an example of liver injury where the hepatic lobules undergoing regeneration were not directly damaged. Most liver injury, however, occurs throughout the liver and a regenerative response occurs in the area of injury itself. The mechanisms regarding liver regeneration may have similar underlying features in both PH and other kinds of liver injury. To test this, we used the BF rat model, draining the BA pool 24 hours prior to administration of CCl_4_ (Figure 2B). Similar to the PH experiments, serum BA rose after CCl_4_ injury, though with a later peak (Figure 7A); liver Cyp7a levels were depressed (Figure 7B), and intestine Fgf15 levels changed little (Figure 7C). In rats with BF, the rise in serum BA after CCl_4_ was significantly diminished though surprisingly the initially elevated liver Cyp7a levels were significantly repressed early after injury, similar to what was seen after PH. Serum ALT, a marker of hepatocyte death, rose significantly 24 hours after CCl_4_, but was not different between controls and rats with depleted BA pools (Figure 7D). Serum bilirubin levels where likewise similar (data not shown). However, there were significantly fewer Ki-67 positive hepatocytes seen at 48 hours after CCl_4_ intoxication (Figure 7E and 7F). These data suggest that in a model of CCl_4_ intoxication, liver injury itself is not affected by the presence or absence of BA, but that BA are required for the normal regenerative effect that follows toxic liver injury.

## Discussion

Liver regeneration is unlike tissue regeneration in other organs in non-human species, and has also been termed “compensatory proliferation.” The proliferation is thought to “compensate” for some function the diminished hepatocyte mass cannot perform, though the specific function yet eludes researchers. Though many secondary signals that govern the compensatory proliferation are known, knowledge of the primary physiological signal(s) would significantly advance the field.

Given that the liver has a finite capacity to secrete BA [22], it is not surprising that after PH the remnant liver cannot secrete the largely unchanged BA pool returning from the intestine. This inability of the remnant liver to secrete the returning BA pool manifests as spillage of portal blood BA into the systemic circulation, with a rise in systemic BA levels minutes after PH. Liver regeneration after PH is at least in part governed by humoral factors [36] and is preceded by signaling events which occur within minutes after PH [37]. BA flux after PH thus fulfills several criteria that make it an attractive physiological candidate for instigation and control of liver regeneration: (1) the hepatic function of BA circulation cannot be maintained by the remnant post-PH liver, (2) abnormal BA flux is evident within minutes after PH, (3) elevated systemic BA after PH are a potential humoral signal for liver regeneration, and (4) the BA pool is a fixed size in relationship to size of the organism, and secretion of this fixed size BA pool could be a governing factor in liver size (and setting of the “hepatostat”). In addition, several studies have implicated BA receptors in liver regeneration, including FXR [13], [14], Fgf15 [15] and TGR5 [16].

The present study sought to clarify the role of BA in liver regeneration after PH, with emphasis on the physiological and signaling dynamics of BA flux. As we have shown, the EHC of BA is significantly disturbed within minutes of PH in the mouse (and rat, earliest timepoint 5 minutes, data not shown), with a relative redistribution of the BA pool from the intestine into the systemic blood. Prevention of this phenomenon through drainage of the BA pool effectively attenuates the BA overload after PH. Prevention of BA return through the portal blood after PH significantly diminishes hepatocyte proliferation as well as regrowth of the liver, and thus clearly links an important physiological liver function to liver regeneration. We found that the effects on liver regeneration are specific for BA by replacing BA in the rat biliary fistula model, as well as by using mice deficient in the intestine BA-specific transporter Asbt (Asbt-/-).

Multiple analyses confirm a lack of normal liver regeneration when BA are not returning to the remnant liver. Complete RNA sequencing of liver tissue after PH in control rats, those with BF and those with BA replacement after BF shows that the genetic pathways of DNA replication are markedly decreased when BA are removed, and is restored when BA are added back to the system. Serum levels of HGF are diminished in the absence of BA, and restored when BA are replenished. Serum IL-6 levels, however, are relatively unchanged in the absence of BA after PH. These findings support the growing body of literature that finds HGF indispensible for liver regeneration, while IL-6, which clearly rises after PH, may not be necessary for liver regeneration specifically. STAT3, one of the primary effectors in the IL-6 signaling pathway, was little affected by the absence of BA after PH (Figure S3A).

PH is followed within minutes by activation of the mitogen-activated kinase JNK, and inhibition of JNK diminishes cyclin D1 expression and liver regeneration [30]. A kinase upstream of JNK and AP-1 signaling, MKK4, was found to be crucial for liver regeneration [38]. BA activate JNK in hepatocytes [32], [39], [40] and such activation is thought to be one of the underlying mechanisms for inhibiting BA synthesis through Cyp7a repression. Our study showed a significant upregulation in AP-1 gene set activation (major transcription factor activated by JNK) 4 hours after PH in control rats. The AP-1 gene set upregulation was significantly attenuated in rats with absent BA, and restored when BA were added back to the model. This finding suggests a mechanistic link between BA signaling and AP-1 mediated liver regeneration.

Others have found that liver size can be increased with enteral BA feeding [13]. Our study validates this finding by showing that liver regrowth after PH is significantly diminished in the absence of BA return to the liver. The molecular mechanisms explaining this phenomenon are unknown, but could involve the Hippo signaling pathway, which is often invoked in regulation of organ size. Indeed, the Hippo pathway gene set is significantly activated at 12 and 24 hours after PH in control rats, but largely inactive when BA are removed, a finding which again can be reversed by replacement of BA.

Despite the findings in this study showing the BA are clearly necessary for normal liver regeneration, it is also clear that BA flux is not the only factor which controls this process. While diminished, hepatocyte proliferation and liver re-growth still occur after PH in the absence of BA return from the intestine, both in the rat bilary fistula and mouse Asbt-/- models. This data supports the notion as suggested by others that multiple pathways govern liver regeneration, but did not determine whether BA secretory capacity of the liver controls liver size and thus the “hepatostat.”

The primary rate-controlling enzyme for BA synthesis, Cyp7a, was markedly down-regulated after PH both in the rat biliary fistula and mouse Asbt-/- models. This finding is surprising, as the two major mechanisms described to down-regulate Cyp7a, increased intestinal Fgf15 or elevated BA causing JNK activation, are both eliminated in the present study, yet the Cyp7a is still profoundly decreased soon after PH. Previous studies have noted the Cyp7a repression after PH [41] and attributed this to HGF and JNK signaling, though blockade of these pathways after PH did not fully account for the Cyp7a repression. As well, Cyp7a repression after PH occurs in FXR-/- mice [13], demonstrating that the repression is FXR-independent. The present study supports and expands this concept, and suggests that an as-yet unidentified signal is responsible for Cyp7a repression after PH, unrelated to high BA levels either in the portal blood or intestine. Identification of the mechanism by which Cyp7a is repressed after PH may therefore point toward a more proximal signal regulating liver regeneration and thus deserves further study.

Finally, our study demonstrates that liver injury (CCl_4_) other than PH also requires BA for liver regeneration. It has long been speculated that any liver injury results later in regeneration, but it is controversial whether the same signals are at play in different injury models. In the present work, however, bile acid flux after CCl_4_ in controls and BF rats looked remarkably similar to that following PH (though delayed by about a day). Indeed, the same profound Cyp7a repression was seen even in the absence of BA return to the liver and virtually absent intestinal Fgf15. These data suggest that, at least in the CCl_4_ acute intoxication model, the contribution of bile acid flux to liver regeneration is similar to that seen after PH.

In sum, our study for the first time describes the physiology of BA flux after PH and its attendant signaling in the liver and intestine. The data clearly ties the function of BA secretion by the liver to hepatocyte proliferation and liver growth after PH, and suggests that BA-mediated JNK and AP-1 activation may provide the mechanistic link between BA flux and hepatocyte proliferation. We further show that Cyp7A repression occurs after PH in the absence of BA overload or elevated Fgf15, and speculate that further investigation of Cyp7a control may lead to earlier signals governing liver regeneration. And while it is clear that BA flux is necessary for normal liver regeneration, it is also clear that other mechanisms are at work, and further research is needed to define other physiological functions of the liver that stimulate the compensatory proliferation.

## Supporting Information

Figure S1 (TIF)

Figure S2 (TIF)

Figure S3 (TIF)

Figure S4 (TIF)

Figure S5 (TIF)

File S1 Contains Tables S1–S3. Table S1. qPCR Primer List. Table S2. Gene set constituents. Table S3. RNA Sequencing Results.
(DOCX)

Materials and Methods S1 (DOCX)
