# Supplementary figures and images for: Bile Acid Flux Is Necessary for Normal Liver Regeneration
Source: PLoS One. 2014 May 19;9(5):e97426. doi: 10.1371/journal.pone.0097426 (PMC4026228; doi:10.1371/journal.pone.0097426)

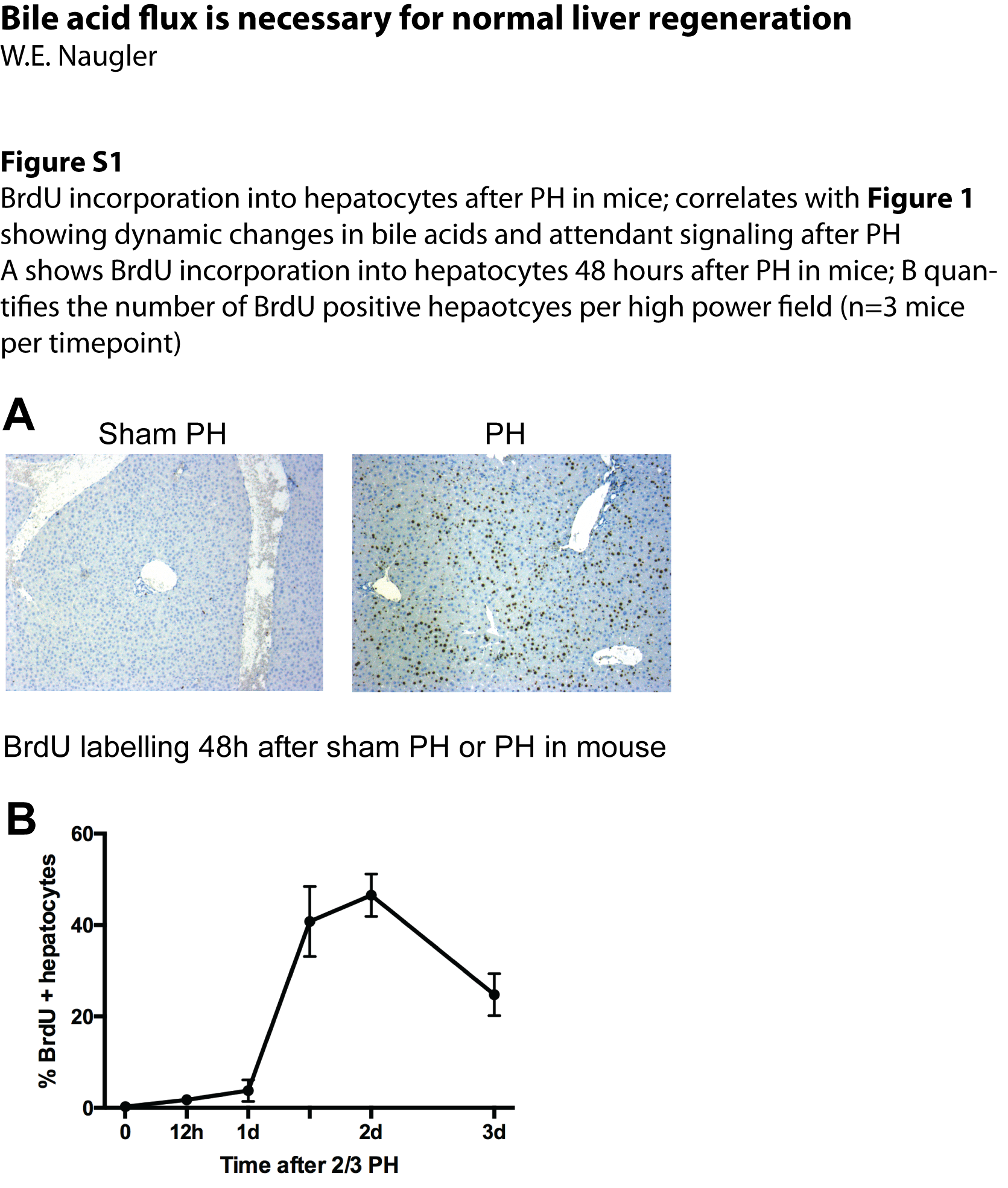

Supplement: Figure S1 — (TIF) [file pone.0097426.s001.tif]

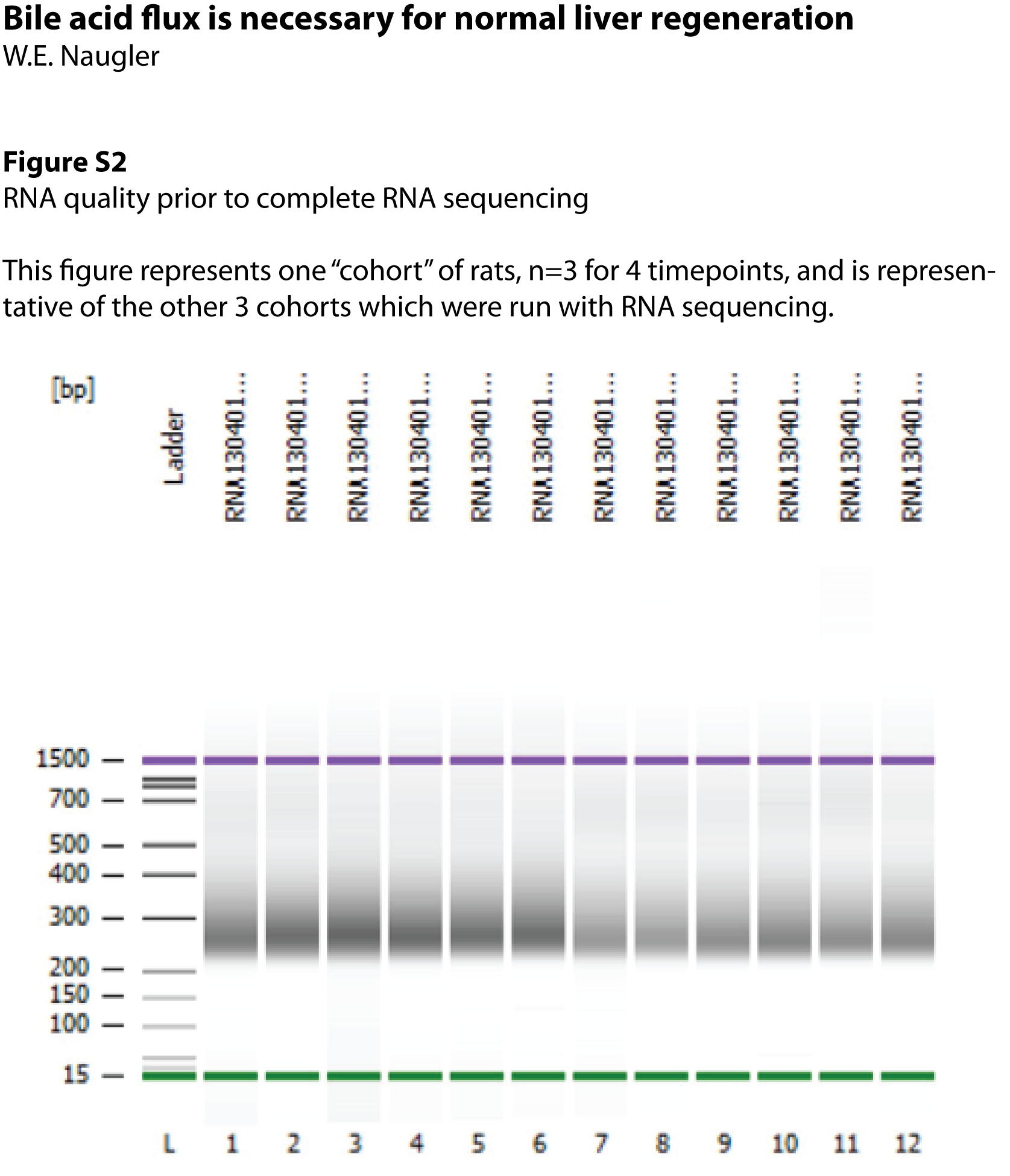

Supplement: Figure S2 — (TIF) [file pone.0097426.s002.tif]

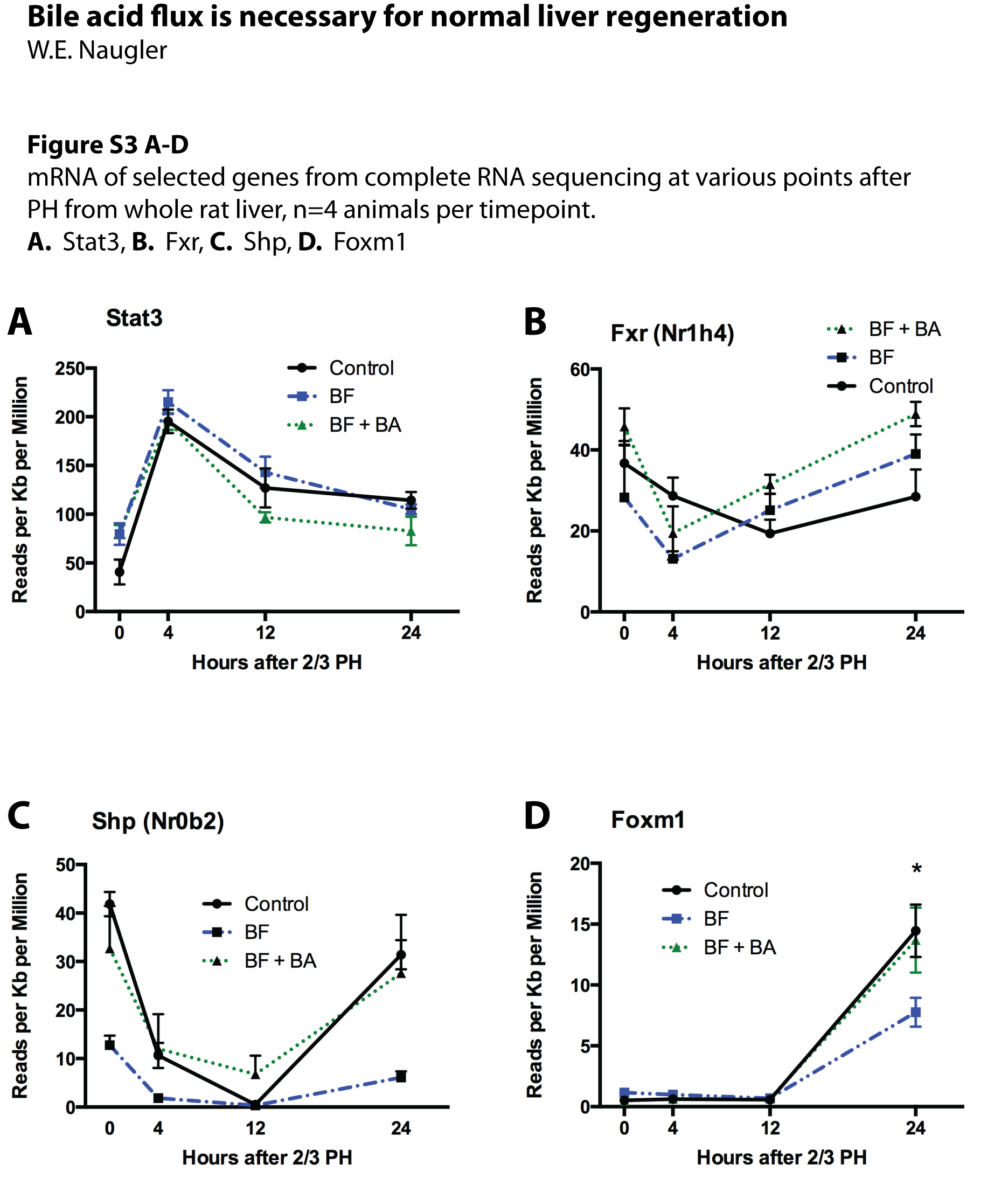

Supplement: Figure S3 — (TIF) [file pone.0097426.s003.tif]

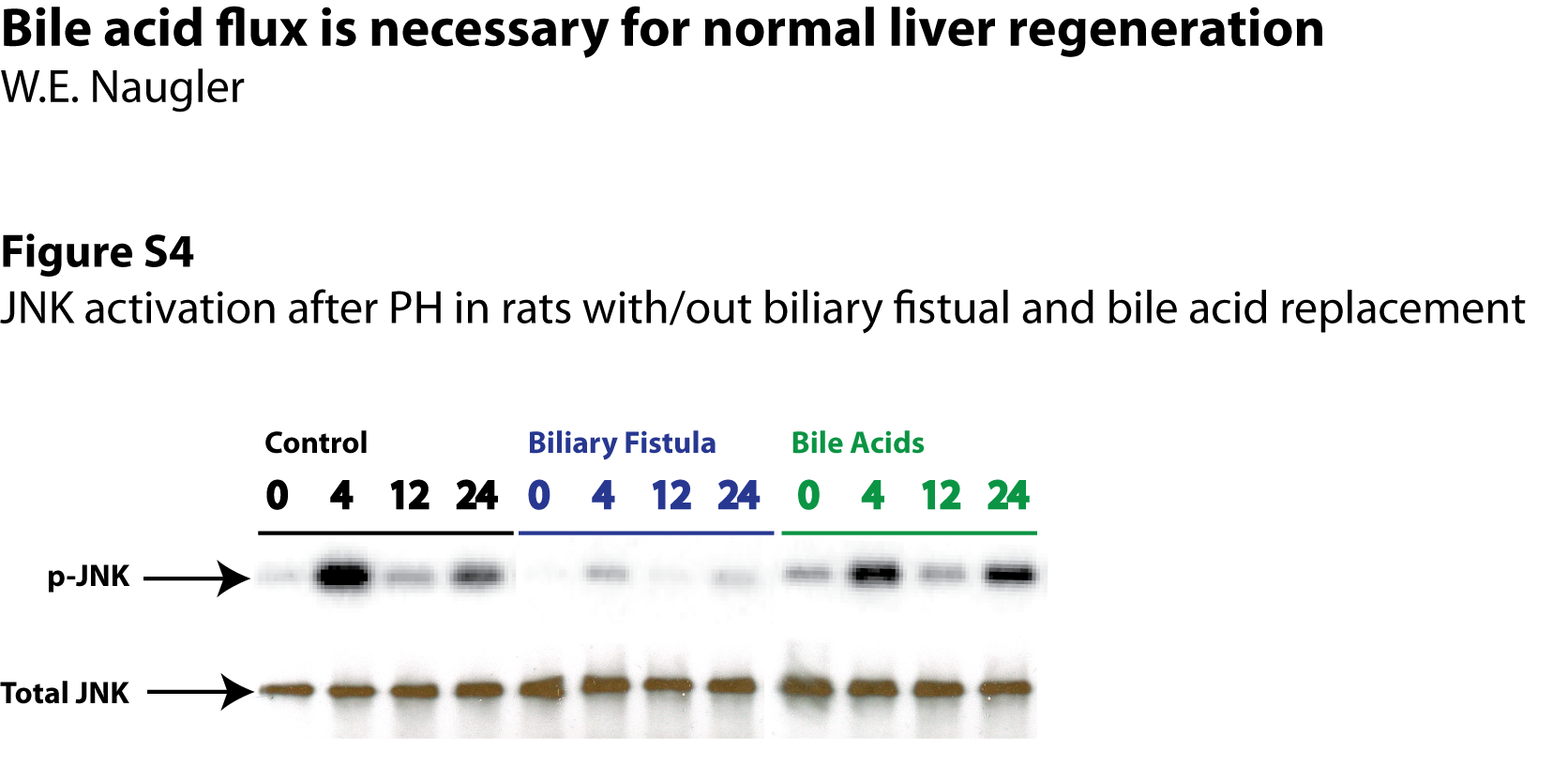

Supplement: Figure S4 — (TIF) [file pone.0097426.s004.tif]

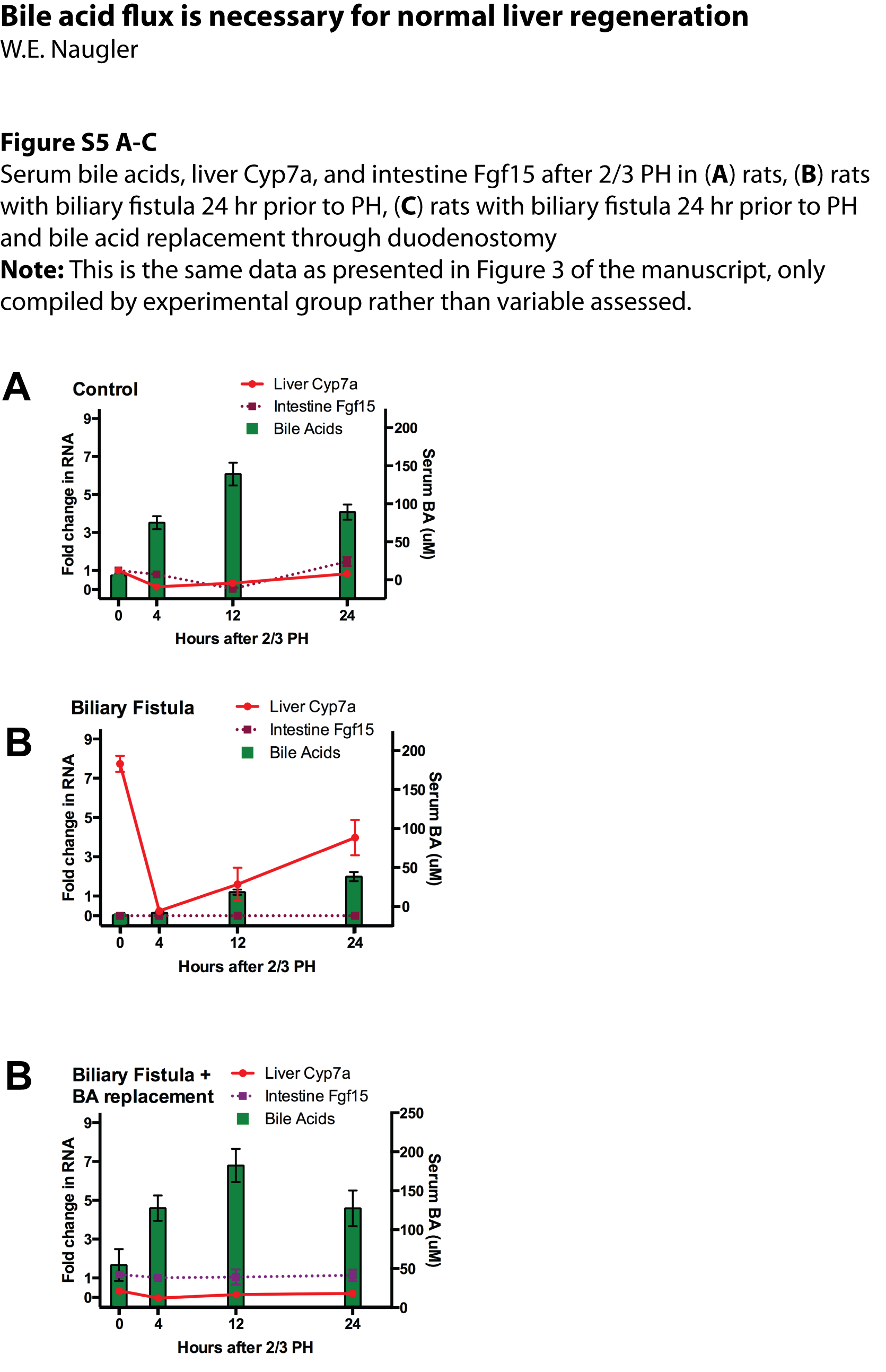

Supplement: Figure S5 — (TIF) [file pone.0097426.s005.tif]
