# Supplementary material for: Bile Acid Flux Is Necessary for Normal Liver Regeneration
Source: PLoS One. 2014 May 19;9(5):e97426. doi: 10.1371/journal.pone.0097426 (PMC4026228; doi:10.1371/journal.pone.0097426)
Supplement: File S1 — Contains Tables S1–S3. Table S1. qPCR Primer List. Table S2. Gene set constituents. Table S3. RNA Sequencing Results. (DOCX) [file pone.0097426.s006.docx]

**Supplemental Tables**

*Table S1* **qPCR Primer List**

| **qPCR primer** | **Sequence (5’-3’)** |
| --- | --- |
| Mouse Cyp7a F | TGGAATAAGGAGAAGGAAAGTA |
| Mouse Cyp7a R | TGTGTCCAAATGCCTTCGCAGA |
| Mouse FGF15 F | GAGGACCAAAACGAACGAAATT |
| Mouse FGF15 R | ACGTCCTTGATGGCAATCG |
| Mouse Gapdh F | CGACTTCAACAGCAACTC |
| Mouse Gapdh R | GTAGCCGTATTCATTGTCAT |
| Rat Cyp7a F | CTGTCATACCACAAAGTCTTATGTCA |
| Rat Cyp7a R | ATGCTTCTGTGTCCAAATGCC |
| Rat FGF15 F | CTCCTCGGTACTGCAAGAAACC |
| Rat FGF15 R | TGTAGCCCAAACAGTCCATTTCCT |
| Rat PCNA F | ATCTAGACGTCGCAACTCCG |
| Rat PCNA R | GCTGCACTAAGGAGACGTGA |
| Rat Gapdh F | GTATCGGACGCCTGGTTAC |
| Rat Gapdh R | ACTGGAACATGTAGACCATGTAGTT |

*Table S2* ***Gene set constituents***

| **DNA Replication** | |  |  |  |  |  |  |
| --- | --- | --- | --- | --- | --- | --- | --- |
| AHCTF1 | CENPC1 | GINS2 | MCM6 | PMF1 | PSMA4 | PSMD12 | RPA1 |
| APITD1 | CENPH | GINS4 | MCM7 | POLA1 | PSMA5 | PSMD13 | RPA2 |
| AURKB | CENPI | GMNN | MCM8 | POLA2 | PSMA6 | PSMD14 | RPA3 |
| B9D2 | CENPK | GORASP1 | MIS12 | POLD1 | PSMA7 | PSMD2 | RPA4 |
| BIRC5 | CENPL | INCENP | MLF1IP | POLD2 | PSMA8 | PSMD3 | RPS27 |
| BUB1 | CENPM | ITGB3BP | NDC80 | POLD3 | PSMB1 | PSMD4 | RPS27A |
| BUB1B | CENPN | KIF18A | NDEL1 | POLD4 | PSMB10 | PSMD5 | RPS27AP11 |
| BUB3 | CENPO | KIF20A | NSL1 | POLE | PSMB2 | PSMD6 | SEC13 |
| CASC5 | CENPP | KIF23 | NUDC | POLE2 | PSMB3 | PSMD7 | SEH1L |
| CCDC99 | CENPQ | KIF2A | NUF2 | PPP1CC | PSMB4 | PSMD8 | SGOL1 |
| CCNA1 | CENPT | KIF2B | NUP107 | PPP2CA | PSMB5 | PSMD9 | SGOL2 |
| CCNA2 | CKAP5 | KIF2C | NUP133 | PPP2CB | PSMB6 | PSME1 | SKA1 |
| CDC20 | CLASP1 | KNTC1 | NUP37 | PPP2R1A | PSMB7 | PSME2 | SKA2 |
| CDC45 | CLIP1 | LIG1 | NUP43 | PPP2R1B | PSMB8 | PSME4 | SKA2L |
| CDC6 | DBF4 | LOC645084 | NUP85 | PPP2R5A | PSMB9 | PSMF1 | SMC1A |
| CDC7 | DNA2 | LOC652826 | ORC1 | PPP2R5B | PSMC1 | RAD21 | SMC3 |
| CDCA8 | DSN1 | MAD1L1 | ORC2 | PPP2R5C | PSMC2 | RANBP2 | SPC24 |
| CDK2 | E2F1 | MAD2L1 | ORC3 | PPP2R5D | PSMC3 | RANGAP1 | SPC25 |
| CDKN1A | E2F2 | MAPRE1 | ORC4 | PPP2R5E | PSMC4 | RB1 | STAG1 |
| CDKN1B | E2F3 | MCM10 | ORC5 | PRIM1 | PSMC5 | RCC2 | STAG2 |
| CDT1 | ERCC6L | MCM2 | ORC6 | PRIM2 | PSMC6 | RFC2 | TAOK1 |
| CENPA | FBXO5 | MCM3 | PAFAH1B1 | PSMA1 | PSMD1 | RFC3 | UBA52 |
| CENPC1 | FEN1 | MCM4 | PCNA | PSMA2 | PSMD10 | RFC4 | XPO1 |
| CENPH | GINS1 | MCM5 | PLK1 | PSMA3 | PSMD11 | RFC5 | ZW10 |
|  |  |  |  |  |  |  | ZWILCH |
|  |  |  |  |  |  |  | ZWINT |

*TableS 2* ***Gene set constituents***

| **AP-1 Acitvation** | **Hippo**  **Pathway** | **Bile Acid Synthesis** |
| --- | --- | --- |
| ATF2 | AMOT | ABCB11 |
| FOS | AMOTL1 | ACOT8 |
| JUN | AMOTL2 | ACOX2 |
| MAPK1 | CASP3 | AKR1C4 |
| MAPK10 | DVL2 | AKR1D1 |
| MAPK11 | LATS1 | AMACR |
| MAPK14 | LATS2 | BAAT |
| MAPK3 | LOC440917 | CH25H |
| MAPK8 | LOC646561 | CYP27A1 |
| MAPK9 | MOB1A | CYP39A1 |
|  | MOB1B | CYP46A1 |
|  | NPHP4 | CYP7A1 |
|  | SAV1 | CYP7B1 |
|  | STK3 | CYP8B1 |
|  | STK4 | HSD17B4 |
|  | TJP1 | HSD3B7 |
|  | TJP2 | SCP2 |
|  | WWC1 | SLC27A2 |
|  | WWTR1 | SLC27A5 |
|  | YAP1 |  |
|  | YWHAB |  |
|  | YWHAE |  |

*Table S3* ***RNA Sequencing Results***

All of the RNA sequencing data used in this study is available at the NCBI GEO website, Series record GSE54673, <http://www.ncbi.nlm.nih.gov/geo/query/acc.cgi?acc=GSE54673>. Each column (example: **Cont-T0-A**) refers to one condition and one time point (**Cont-T0-A** refers to Control rat liver collected at the time of PH), but includes data from 4 animals which has been averaged according to RPKM count).
