# Supplementary material for: Bile Acid Flux Is Necessary for Normal Liver Regeneration
Source: PLoS One. 2014 May 19;9(5):e97426. doi: 10.1371/journal.pone.0097426 (PMC4026228; doi:10.1371/journal.pone.0097426)
Supplement: Materials and Methods S1 — (DOCX) [file pone.0097426.s007.docx]

**Supplemental Materials and Methods**

*Quantitative real-time RT-PCR (qPCR)*

RNA was isolated, reverse transcribed, and measured by real-time reverse

transcriptase–PCR using SYBR green as previously described.[^1^](#_ENREF_1) (Life Technologies,

Carlsbad, CA) (primers are listed in **Table S1**).

*Histology*

Rat and mouse liver was immersed in 4% formalin immediate after collection, embedded in paraffin, and slides cut in the usual fashion. Routine hematoxylin and eosin staining was done for all samples, while separate slides were used for BrdU staining. Rat staining was performed with rat mAB OBTOO3OG (Accurate) for Brdu or mAb YSRTMCA289 (Accurate) for Ki-67 and visualized with BioGenex AEC. Sections were counterstained lightly with hematoxylin. Mouse sections were stained with mAB AB125306 (Abcam) with similar processing.

*Complete RNA sequencing*

Sequencing libraries were constructed using the Illumina TruSeq RNAseq kit. Briefly, poly(A)+ RNA was isolated from total RNA using oligo-dT coated magnetic beads. (See **Figure S2** for RNA quality control prior to library creation). The recovered RNA was then chemically fragmented. First strand cDNA was generated using random hexamers as primers for reverse transcriptase. Following second strand synthesis, the ends of the double stranded fragments were repaired and then a single “A” nucleotide was added to each end. Illumina adaptors were ligated to the cDNAs. Limited round PCR was used to amplify the material to yield the final library. Library concentration was determined using real time PCR with primers complementary to the Illumina adaptors. Sample libraries were diluted and applied to an Illumina paired end flow cell at a concentration appropriate to generate about 180 million reads per lane. All libraries were prepared with indexing barcodes to permit multiplex runs. 50 cycle single read sequencing were used to generate base call files. Illumina’s CASAVA package was used to assemble the reads into standard fastq formatted data.

*RNA sequencing gene set analysis*

Raw fastq data from RNA libraries was transformed into Reads Per Kilobase Million (RPKM), which allows a normalized comparison between libraries. RPKM data was analyzed using Gene Set Enrichment Analysis (GSEA) as described.[^2^](#_ENREF_2) Gene set constituents are noted in **Table S2**). Differences in gene sets were considered significant if the false discovery rate (FDR) qvalue was < 0.05, a more stringent statistic than the p value. All differences between gene sets with an FDR qvalue < 0.05 also had p values < 0.05. Complete RNA sequencing data can be found at the NCBI GEO website, Series record GSE54673, <http://www.ncbi.nlm.nih.gov/geo/query/acc.cgi?acc=GSE54673>.

*Serum Bile Acid (BA), ALT, HGF, and IL-6 determinations*

Serum total BA concentrations were performed in triplicate with an enzymatic Total Bile Acids Test Kit (Diazyme, Cat # DZ042A-K) according to the manufacturer’s instructions. Serum ALT levels were assayed in triplicate with an ALT (SGPT) Reagent Kit (Color Endpoint) (Bio-quant, Cat # BQ 004A-CR). Serum HGF was determined with the Mouse/Rat HGF Quantikine ELISA kit (R&D Systems, Cat # MHG00), and serum IL-6 levels assayed with the Rat IL-6 Quantikine ELISA Kit (R&D Systems, Cat # R6000B).

1. Madison, B.B.*, et al.* Cis elements of the villin gene control expression in restricted domains of the vertical (crypt) and horizontal (duodenum, cecum) axes of the intestine. *J Biol Chem* **277**, 33275-33283 (2002).

2. Subramanian, A.*, et al.* Gene set enrichment analysis: a knowledge-based approach for interpreting genome-wide expression profiles. *Proc Natl Acad Sci U S A* **102**, 15545-15550 (2005).
